# An Update on Photodynamic Therapy of Psoriasis—Current Strategies and Nanotechnology as a Future Perspective

**DOI:** 10.3390/ijms23179845

**Published:** 2022-08-30

**Authors:** Sebastian Makuch, Mateusz Dróżdż, Alicja Makarec, Piotr Ziółkowski, Marta Woźniak

**Affiliations:** 1 Department of Clinical and Experimental Pathology, Wroclaw Medical University, 50-368 Wroclaw, Poland; 2 Laboratory of RNA Biochemistry, Institute of Chemistry and Biochemistry, Freie Universität Berlin, Takustraße 6, 14195 Berlin, Germany; 3 Faculty of Biotechnology, University of Wroclaw, 50-383 Wroclaw, Poland

**Keywords:** photodynamic therapy, psoriasis, nanotechnology, PDT

## Abstract

Psoriasis (PS) is an immune-mediated skin disease with substantial negative effects on patient quality of life. Despite significant progress in the development of novel treatment options over the past few decades, a high percentage of patients with psoriasis remain undertreated and require new medications with superior long-term efficacy and safety. One of the most promising treatment options against psoriatic lesions is a form of phototherapy known as photodynamic therapy (PDT), which involves either the systemic or local application of a cell-targeting photosensitizing compound, followed by selective illumination of the lesion with visible light. However, the effectiveness of clinically incorporated photosensitizers in psoriasis treatment is limited, and adverse effects such as pain or burning sensations are frequently reported. In this study, we performed a literature review and attempted to provide a pooled estimate of the efficacy and short-term safety of targeted PDT in the treatment of psoriasis. Despite some encouraging results, PDT remains clinically underutilized. This highlights the need for further studies that will aim to evaluate the efficacy of a wider spectrum of photosensitizers and the potential of nanotechnology in psoriasis treatment.

## 1. Introduction

Psoriasis is an immune-mediated and chronic skin disease characterized by the abnormal proliferation of skin cells, known as keratinocytes. It is estimated that its prevalence varies from 0.14% (East Asia) to 5.32% (Central Europe) [1]. This disease is associated with genetic predisposition, autoimmune disorders, mental health, and environmental factors, including infections, stress, alcohol, smoking, obesity, physical trauma, or certain medications, such as lithium and beta-blockers [2,3]. Its pathogenesis is defined as a multi-factorial process that depends on uncontrolled increased proinflammatory cytokine expression, among the most important being IL-17, IL-21, IL-22, IL-23, and IL-26. For instance, IL-17 and IL-23 stimulate keratinocyte proliferation and increase the secretion of TNF-α and chemokines, which enhance the activation of dendritic cells, leading to inflammation [4,5,6].

For many years, psoriasis has been treated by many traditional approaches, including acupuncture, hydrotherapy, and dietary treatment, which can help in controlling the progress of the disease and alleviating some of the symptoms. Scientists have implemented a wide range of therapeutic tools customized according to the type of psoriasis, its location, extent, and severity, including corticosteroids, calcipotriene, coal tar, oral systemics (e.g., acitretin, cyclosporine, methotrexate), biologics (etanercept, infliximab, alefacept, efalizumab, and ustekinumab), as well as phototherapy, with the use of ultraviolet B light (UVB), psoralen ultraviolet A light (PUVA), or photodynamic therapy (PDT) [7,8].

In contrast to biological agents and other drugs, phototherapy is an effective and safe method that does not cause any systemic side effects. This technique was originally developed with the idea of using broadband ultraviolet B light (BB-UVB, 290–230 nm). However, subsequent studies proved the better efficacy of the narrow-band ultraviolet B (NB-UVB, 311 nm), and even an excimer laser/lamp (308 nm) used as a monochromatic UVB source [9]. All these methods are currently used as first-line therapies for stable plaque psoriasis. The next key achievement turned out to be the development of psoralen ultraviolet A (PUVA) treatment, which combines a photosensitizing drug (PS) and UVA radiation. This method is currently the first-line therapy for refractory psoriatic plaques [10]. The other light source that proved to be efficient in psoriasis treatment was the flash lamp pumped pulsed dye laser (585–595 nm), which is preferred for nail psoriasis [2]. Nevertheless, because of its efficacy and cost-effectiveness, PDT seems to be an attractive option for psoriasis treatment [7]. This technique is based on a photochemical reaction used to destroy diseased cells selectively. It comprises three elements: a light-sensitive dye (photosensitizer), which accumulates in the intracellular compartment of the cells, a light with an appropriate wavelength that is capable of exciting the photosensitizer, and oxygen dissolved in the tissue being treated. Target sites for PS include different organelles, such as the plasma membrane, mitochondria, lysosomes, Goldi apparatus, or endoplasmic reticulum [11]. Under specific conditions, PS is activated by absorbing the photons of light energy and transferring them to molecular oxygen, leading to the formation of other reactive oxygen species (ROS), including hydroxyl radicals (OH) or superoxides (O^2^). These compounds produce strong reactivity with lipids, nucleic acids, proteins, and other biochemical substrates, leading to the activation of pathogenetic mechanisms, which have been thoroughly described in various reviews [8,12,13]. Photodamage leads to cell death through apoptosis, necrosis, or autophagy, depending on the cell type, PDT dosimetry (light intensity), and PS type. Photosensitizers localized in the mitochondria affect apoptosis after irradiation, whereas those in the plasma membrane stimulate necrosis [14]. However, the effectiveness of clinically incorporated photosensitizers is limited, and adverse effects such as pain or burning sensations are frequently reported [15,16]. Therefore, it is desirable to develop novel photosensitizers with a convincing safety profile and potent, selective activity. 

In this study, we aimed to review the literature in order to provide a pooled estimate of the efficacy and short-term safety of targeted PDT in the treatment of psoriasis. We focused on describing clinical trials evaluating photosensitizers in the PDT of psoriasis. Due to significant disagreement on the use of this method in the scientific community, our goal is to estimate whether PDT treatment is worth further consideration for psoriasis treatment. Reported differences may be explained by variables in terms of patient selection criteria, drug concentrations, the light spectrum, or dosages [17]. Therefore, such a juxtaposition of the latest achievements in this regard seems to be very beneficial for further analysis.

## 2. Efficacy of ALA-PDT Therapy

The use of PDT in psoriasis treatment is subject to many discussions. To the best of our knowledge, there has been no study showing a 100% recovery of psoriasis PDT-treated patients. Nevertheless, several studies showed the prominent role of PDT in blocking the uncontrolled production of inflammatory cytokines that lead to the apoptosis of T-lymphocytes and inflammation during psoriasis development [18,19].

One major advancement in the use of PDT in psoriasis treatment was the development of 5-aminolevulinic acid (ALA) as a photosensitizing agent, localizing in the abnormal epithelium and causing cytotoxic effects [20]. In physiological conditions, ALA is an early intermediate in the heme biosynthesis pathway. However, this compound can be further metabolized by the cells into protoporphyrin IX (PpIX), which may be subsequently activated by visible light [21]. The accumulation of PpIX in the intracellular compartment of the cells results from the lack of conversion to heme because of the limiting enzyme known as ferrochelatase. This condition leads to oxidative damage and induces cytotoxicity. Various reviews have extensively analyzed the metabolic pathways of ALA and its derivatives in PDT therapy [21,22,23].

Including the clinical trials, ALA-PDT was first mentioned in 1994, when three patients with chronic plaque-stage psoriasis were administered 10% ALA. The clearance of the psoriatic lesion with PDT was comparable to those treated with dithranol; all patients experienced a mild burning sensation [24]. This study was further continued after four years on a larger study group of 22 patients with chronic plaque psoriasis. Among 80 treatment sites, there was a clear reduction in 14 sites (18%), and a 30–50% reduction in the scale, erythema, and induration (SEI) index. There was no improvement observed in 60 sites (75%) [25]. Following this study, many other clinical trials described some beneficial effects of ALA-PDT in psoriasis and found that the apoptosis of T-lymphocytes within inflammatory plaques was associated with reduced inflammatory cytokine production (such as TNF- α, IL-1β, and IL-6) and the normalization of keratinocyte proliferation. The potential mechanism of PDT-action was described recently by Wang et al. who found that ALA-PDT excites the MAPK pathway, promoting the expression of p38, JNK, and ERK kinases [26]. This activation leads to the upregulation of the apoptotic genes PARP and caspase 3, which enhance cell apoptosis [24]. Furthermore, Yi et al. showed the role of SOCS1 and SOCS3 in ALA-PDT-mediated psoriasis treatment. ALA-PDT activates SOCS1/3 productivity by increasing the intracellular oxidative stress in keratinocytes. Therefore, SOCS1/3, which is known as a potential blocker of the JAK pathway, attenuates the proliferation of keratinocytes in psoriasis [27]. In another in vitro study, Chen et al. demonstrated that ALA-PDT reduced the number of abnormal T cells and the mRNA expression of IL-17 and IFN-γ involved in a psoriasis lesion [28]. Nevertheless, information on the molecular mechanism of ALA-PDT action in psoriasis treatment is still scarce and requires further investigation.

Considering the recently published studies, the use of systemic ALA-PDT for psoriasis was also addressed by Liu et al., who described the case of a 49-year-old male patient with chronic plaque psoriasis. The patient had a psoriasis lesion on his finger with pyogenic granuloma (PG). After one week of ALA-PDT treatment, signs of improvement were demonstrated. There were also no signs of reoccurrence after one month of treatment [29]. However, although this case shows the treatment success of ALA-PDT, it relates only to one patient, which may be not sufficient to statistically estimate the efficiency of ALA-PDT. Other clinical researchers noticed a quite low efficiency of ALA-PDT. In independent studies, Le Pillouer-Prost et al., Almutawa et al., and Choi et al. found that due to the variability in clinical responses and severe pain, ALA-PDT was not sufficient for chronic plaque psoriasis [15,16,30]. The most common side effect experienced with topical ALA-PDT was pain at the irradiation site, which lasted up to 2 days in some patients [31]. The pain was described as a stinging or burning sensation in the treatment sites, sometimes leading to discontinuation of treatment with this method. Moreover, low photodynamic doses (5–10 J/cm^2^) and topical anesthetics such as 3% lidocaine hydrochloride cream, capsaicin cream, or lidocaine-prilocaine 5% cream (EMLA) turned out to be ineffective in alleviating the pain caused by ALA-PDT [32]. Taken together, these findings present a major challenge for researchers to improve the efficacy of ALA-PDT in psoriasis treatment. The solution seems to be the development of novel photosensitizers or using PDT in combination with other techniques (e.g., nanotechnology); this concept is discussed later in this review. 

Considering the fact that ALA-PDT has more advantages than chemotherapy or radiation (presenting little or no systemic toxicity) and surgery (healing without scarring) to treat psoriasis, this method has significant potential for improvement [33]. Maytin et al. performed a pilot study assessing the use of vitamin D (VD) combined with ALA-PDT. The concept appeared due to the common prescription of vitamin D analogs (such as calcitriol and calcipotriol) against psoriasis by dermatologists. Seven patients with chronic plaque psoriasis were enrolled in this study; each plaque was treated with topical 0.005% calcipotriol (cream or ointment) or a matched vehicle control applied twice daily for 6 days. On day 7, patients underwent one PDT session with topical 20% ALA, red or blue light, and a power density of 100 mW/cm^2^. In findings inconsistent with the researchers’ hypothesis, no clinical improvement in psoriasis was observed for plaques treated with calcipotriol cream. However, in the calcipotriol ointment-treated plaques, clinical success became apparent due to VD preconditioning. A psoriasis symptom inventory (PSI) index revealed a greater decline on the VD-preconditioned side than on the vehicle control side. Moreover, a remarkable decline in the severity of itching was reported within the VD-pretreated plaques compared to the control plaques. Thus, these observations highlighted the promising potential of ALA-PDT in alleviating psoriatic lesions pretreated with vitamin D ointment [33]. Following this concept, calcipotriol ointment was also administered to the psoriatic area of the leg of a patient suffering from squamous cell carcinoma (SCC). Together with skin dermabrasion and the PDT technique, the patient was successfully treated [34].

Furthermore, it has been shown that 80% of patients with psoriasis exhibit symptoms in their nails [35]. Their involvement is associated with a high prediction of psoriatic arthritis. Therefore, it is crucial to treat nail psoriasis, which is challenging due to therapy resistance. In an open-trial study with 69 nails from 8 patients, Tehranchinia et al. compared the efficacy of clobetasol 0.05% ointment and ALA-PDT in the treatment of severe nail psoriasis. The mean nail psoriasis severity index (NAPSI) scores in the nails treated with clobetasol were significantly less than those in the nails treated with ALA-PDT. However, this finding changed diametrically 6 months after the last treatment session. There was a significant time-effect improvement in all the nail matrix, nail bed, and total NAPSI scores in both treatment groups. Although more data are needed for further evidence, these initial results suggest that the efficacy of ALA-PDT at a 24-week follow-up was greater than that of clobetasol [36]. 

**Figure 1:**
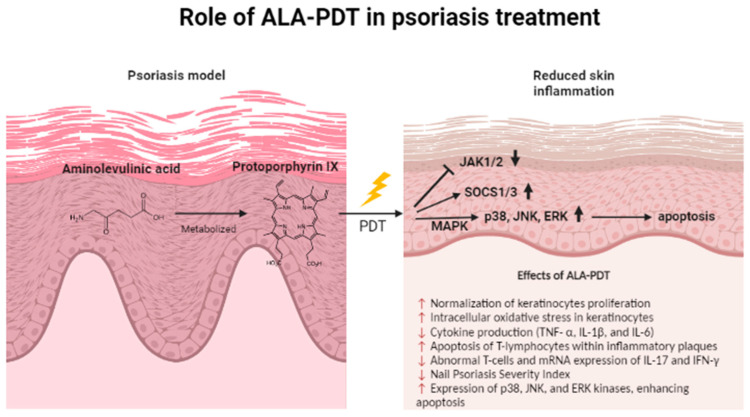
The role of ALA-PDT in psoriasis treatment. Aminolevulinic acid (ALA) is metabolized by cells into protoporphyrin IX (PpIX), which may be subsequently activated by visible light. The effects of ALA-PDT on a psoriasis model and the affected genes have been summarized.

The effects of ALA-PDT on keratinocytes and in vivo model of psoriasis have been summarized and shown in Figure 1.

## 3. Efficacy of Non-ALA-PDT Therapy

To improve selective targeting, localized action, and the stimulation of immune responses, the majority of the current studies aimed to design the most efficient porphyrin-based photosensitizers for PDT. These compounds contain photophysical properties suitable for PDT application, such as the high quantum yield of singlet oxygen and light absorption at 600–750 nm, increasing light penetration to deeper areas of psoriatic lesions [37]. It is already discovered that the number and location of the positively charged groups in the macrocyclic structure play a key role in porphyrin uptake by the target cells and for appreciable ^1^O_2_ production [38]. For instance, Calzavara-Pinton et al. performed a study with a modified version of ALA, known as methyl aminolevulinate (MAL), exhibiting more lipophilic properties. Thanks to this structural change, MAL may penetrate deeper into the skin than ALA. In this retrospective observational study, including 221 patients from 20 Italian hospital centers, 17 of them had psoriatic plaques. Four patients (4/17, 23.5%) developed a marked inflammatory reaction, and 5 patients (5/17; 29.4%) had severe pain and/or a burning sensation. Six (6/17; 35.3%) patients showed marked improvement [17]. Furthermore, Slomp et al. analyzed the photodynamic effect of different porphyrin derivates on HaCaT keratinocytes. They found that those porphyrins presenting at least two adjacent positively charged groups showed the most anti-inflammatory and anti-hyperproliferating effects, leading to the reduction of edema, cellular infiltration, and hyperproliferation of the epidermis [39]. Similarly, an immunosuppressive effect of a porphyrin form (5,10-diphenyl-15,20-di(N-methylpyridinium-4-yl) porphyrin) was recently reported in a mouse psoriasis model. PDT diminished several inflammatory indicators, including proinflammatory cytokine secretion and neutrophil infiltration, and led to reduced keratinocyte proliferation [40]. Another photosensitizer that has been proven to be active against skin inflammation is the α-(8- quinolinoxy) zinc phthalocyanine (ZnPc-F7). This compound is characterized by good solubility and low toxicity. Due to its excitation at 670 nm, it may penetrate more deeply into the skin compared to the PUVA-based treatment. Liu et al. witnessed a significant reduction in HaCaT cell proliferation and IL-17 mRNA expression after ZnPc-F7-mediated PDT, indicating a therapeutic effect in psoriasis [41]. The modified version of phthalocyanine tested in the psoriatic lesion was the silicon phthalocyanine (Pc) 4, coupled with red light. Pc4-PDT elicited cell death through apoptosis on activated CD3+ T cells in the psoriatic lesion, in a dose-response manner [42]. Furthermore, a recent study identified another potential therapeutic candidate for the PDT of psoriasis. Lin et al. investigated the anti-inflammatory effects of novel NNO-tridentate vanadium (IV) complexes in a psoriasis-like skin disease mouse model. These compounds reduced the expression of IL-17 and IL-22 cytokines, suggesting a promising role in relieving psoriatic symptoms [43]. 

While ALA-PDT seems to have been more extensively researched, there is a gap in the knowledge regarding the efficacy of other photosensitizers in psoriasis treatment. Table 1 summarizes the most critical in vivo and clinical studies since the onset of PDT in psoriasis treatment. This table includes all details about the treatment methods, i.e., the method of delivery, wavelength, treatment parameters, and the total number of treatment sessions; all are involved in the final success of a complete recovery. 

## 4. Nanotechnology Combined with PDT in Psoriasis Treatment—Future Perspectives

Over the past few years, one of the most significant prospects in therapeutics is nanotechnology. This field has provided the development of novel nanostructured drug release systems and has enabled the application of novel therapeutic agents against psoriasis. While nanotechnology combined with PDT has been widely developed in other skin disorders, in terms of psoriasis treatment, it has been inadequately researched. Compared with conventional drug delivery systems, the use of nanotechnology allows scientists to modify the solubility of hydrophobic materials, execute the controlled or sustained release of a drug to a specific site, and enhance drug stability, with reduced undesirable effects [55]. Nanocarriers may penetrate the skin, enhancing the concentration gradient at the skin surface, and can provide physical and chemical properties allowing the release of the drug at the delivery site [56]. 

**Figure 2:**
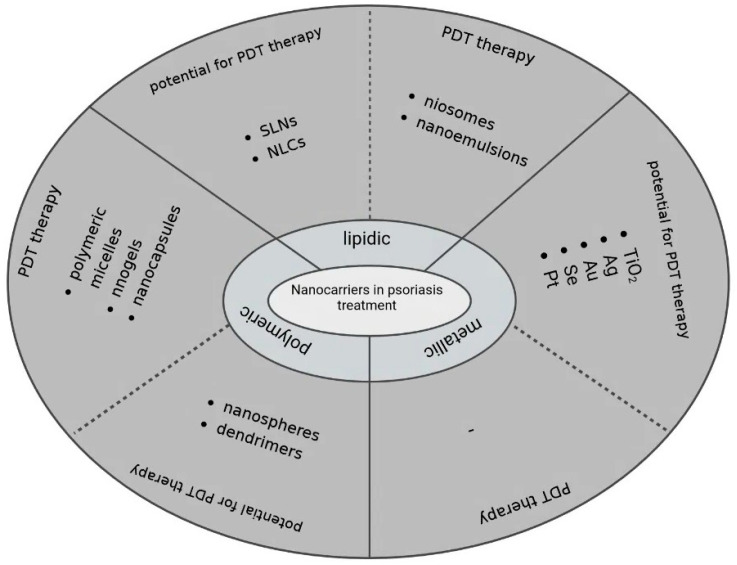
Diagram showing the use of analyzed nanocarriers in psoriasis treatment. Nanocarriers were classified by various studies reporting their use in PDT-based psoriasis treatment and studies proving their great potential in the treatment of other skin diseases.

Several studies have evaluated novel nanocarriers in PDT, including polymeric, lipidic, and metallic nanocarriers. Among the polymer-based nanoparticles, dendrimers, polymeric micelles, nanogels, nanospheres, and nanocapsules have been utilized in the PDT of various skin diseases [57,58]. Therefore, it is reasonable to investigate the role of these nanosystems in psoriasis treatment. Nanocarriers that have been used in PDT of psoriasis and those with high potential have been summarized in Figure 2. 

Dendrimers have gained more interest as nanocarriers over the past decade. They are synthesized from polyfunctional monomers, forming a three-dimensional architecture with unique physicochemical features, such as high reactivity, good solubility, and biocompatibility. Due to their simple modification, the ability to target a delivery site, and guaranteed reproductive pharmacokinetic behavior, dendrimers are widely studied as drug delivery systems for skin disorders. For instance, in 2001, Batah et al. [59] and, eight years later, Casas et al. [60] undertook attempts to encapsulate 5-ALA for dendrimers. These independent studies showed the improvement of 5-ALA stability and its pharmacokinetic profile, suggesting their potential use as macromolecular prodrugs for PDT. Consistently, in 2018, Zhou et al. synthesized a series of iron-chelating agents with ALA conjugated to dendrimers, to improve ALA-PDT efficiency by enhancing PpIX accumulation. Their findings on human cancer cell lines revealed a higher efficacy of PpIX generation in ALA-HPO dendrimers than with ALA alone. This system provides a promising option to enhance the treatment effect of ALA-PDT in cancer [61]. However, the potency of the abovementioned agents has never been tested on psoriasis models.

Polymeric micelles are nanosized molecules that are synthesized from amphiphilic copolymers. Due to their low toxicity, core-shell architecture, nano-size, and relatively high stability, these compounds are often used in drug delivery systems [62]. Considering their use in PDT, Yin et al. designed an amphiphilic zinc phthalocyanine polymer conjugate in the form of a monomolecular micelle. This nanocarrier showed efficient anti-psoriasis activity on psoriasis guinea pig models [63]. To further investigate these findings, zinc phthalocyanine was loaded into nanocapsules, including chitosan nanocapsules, poly-ε-caprolactone (PCL), and PCL-coated chitosan nanocapsules to test their photodynamic activity in psoriasis treatment. These formulations increased skin permeation and drug release rates. All forms of ZnPc conjugated to micelles exhibited a more sustained drug release with no burst effect, suggesting their beneficial role in PDT for psoriasis treatment [64]. 

Nanogels are hydrophilic crosslinked polymeric nanoparticles that are characterized by a high capacity to incorporate molecules and drug encapsulation capacity, as well as tunable size, ease of preparation, and minimal toxicity [65]. Considering PDT, Wang et al. constructed a chitosan/hyaluronan-based nanogel to co-load methotrexate (MTX) and 5-aminolevulinic acid (ALA) for combined chemo-photodynamic therapy for psoriasis. The results showed enhanced penetration and retention of MTX and ALA passing through and into the skin of psoriatic mice [66]. A drug-targeting delivery system with MTX-loaded nanoparticle was also developed by Yin et al., who observed increased cancer cell apoptosis [63]. These results seem promising against psoriatic lesions, due to their potential ability to induce apoptosis in hyperproliferating keratinocytes. Furthermore, Freag et al. synthesized a hydrogel consisting of a liquid crystalline nanoparticulate (LCNP) reservoir of berberine, blended with oleate (Brb-OL). Berberine is one of the most promising agents derived from natural plants for the management of psoriasis. A study revealed a threefold increase in drug accumulation and a tenfold augmentation of drug permeation compared to crude berberine, leading to alleviation of the inflammatory cytokines exhibited in psoriasis [67]. More studies are required to investigate the role of nanogels as drug delivery systems in psoriasis treatment. 

Due to their ability to enhance skin permeation while entrapping pharmaceutical ingredients, nanospheres and nanocapsules are more likely to be tested as topical drug administration systems for psoriasis therapy [68]. These vesicles differ from each other in their morphology and architecture. Nanocapsules contain either an oily or aqueous core, surrounded by a polymeric shell, usually in combination with a mixture of lipophilic and hydrophilic surfactants. In contrast, nanospheres are dense polymer-based matrix systems [69]. Considering their application in the PDT of other skin diseases, Shi et al. synthesized multifunctional hybrid nanospheres that consisted of Fe^3+,^ aggregation-induced emission (AIE) PS and the Bcl-2 inhibitor (sabutoclax). Due to the presence of Fe^3+^, intracellular O_2_ concentration was increased in parallel to the increase in the nanosphere concentration taken up by tumor cells. Both in vitro and in vivo results show the potential of multifunctional hybrid nanospheres in PDT therapy [70]. A promising therapeutic effect in PDT therapy was also shown by Liu et al., who formed biodegradable cancer cell membrane-coated mesoporous copper/manganese silicate nanospheres [71]. In the case of nanocapsules, Amantino et al. developed a nanocapsule containing a poly(lactide-co-glycolide) (PLGA) coating for the encapsulation of an anthraquinone derivative. This nanoparticle increased the drug’s uptake and efficacy, suggesting its promising use in PDT [72].

The first attempts to use nano-sized particles in drug discovery systems were based on lipids. They are natural carriers, which makes them easily available and cost-effective. Among the lipid-based nanoparticles tested in combination with PDT for psoriasis treatment, we can distinguish poly-amphiphiles (such as niosomes), solid lipid nanoparticles (SLNs), and nanostructured lipid carriers (NLCs), as well as nanoemulsions [58]. 

Niosomes are non-ionic surfactant-based vesicles that have been proved to increase the residence time of drugs in the epidermis while reducing systemic absorption [73]. Parnami et al. formulated MTX- and trioxysalen-loaded niosomes and found some beneficial effects while performing experiments on a mouse model. Their application to the epidermis while using narrow-band UV radiation increased the local concentrations of encapsulated drugs and reduced the systemic side effects [74]. Relatively similar findings were reported by Abu et al., who found promising therapeutic effects from acitretin-based niosomes. The developed system inhibited the proliferation of HaCaT cells in vitro and in vivo, showing decreased epidermal thickness without detectable side effects [75]. 

In general, NLCs are very similar to SLNs, both being forms of carriers of hydrophobic drugs that are dispersed within the core of the lipid particles. The only difference is in the structure; NLCs do not have a perfect crystal structure, therefore, they enable better drug encapsulation with less drug leakage. Viegas et al. developed a multifunctional nanostructured lipid carrier (NLC) that allowed the co-delivery of both tacrolimus and siRNA for the TNF-α to the psoriatic plaques. Tacrolimus, as the inhibitor of calcineurin, interacts with some of the cytokines involved in psoriasis pathogenesis, including TNF-α, while antisense therapy using siRNA is one of the more promising strategies to silence the different cytokines and pro-inflammatory molecules involved in psoriasis pathogenesis. This complex showed the high encapsulation efficiency of TAC and effective TNF-α siRNA complexation, providing drug distribution to some extent in the deeper layers of the skin, with low toxicity associated with the uptake of NLC in 4 h, and an approximately 7-fold reduction of TNF-α expression after topical application in psoriatic mice [76]. The co-delivery of other molecules, including drugs and siRNA, through nanocarriers was also evaluated in other studies aiming to control the psoriatic process [77,78]. High encapsulation efficiency was also determined by Wang et al., who constructed N3-labeled cell membrane-derived nanovesicles coated with IR-780-PLGA nanoparticles (N3-NV-INPs). This platform inhibited keratinocyte proliferation and the release of cytokines such as IL-17, IL-22, and TNF-α, indicating its potential in clinical application [79]. Taking into account SLNs, no study has determined their efficacy in PDT-mediated psoriasis treatment. However, Goto et al. have successfully synthesized aluminum chloride phthalocyanine-loaded SLNs and have shown promising signs of application in the treatment of melanoma [80].

Among the emulsion-based nanosystems, nanoemulgels are gaining more and more popularity. These consist of a nanoemulsion that is further incorporated into a water-based gel using different polymers. With its usage, water softens and removes the hyper-keratinized scales formed due to the deposition of the dead cells on the skin that hinder controlled drug release and transport through the skin layers [81]. By a low-energy emulsification method, Algahtani et al. formed a curcumin nanoemugel with increased solubility and skin penetrability in psoriatic mice. Curcumin is a natural compound with inhibitory effects on NF-Kß and MAPK, as well as the STAT3 pathways, and the ability to downregulate the proinflammatory cytokines involved in psoriasis pathogenesis [82]. To further investigate these findings, Gomez et al. determined that curcumin-loaded chitosan/alginate nanoparticles (Cur-CS/Alg NPs), together with a blue light emitting diodes (LED) light irradiation, repressed the hyperproliferation of TNF-α-induced HaCaT cells. Curcumin was also tested in a study conducted by Woźniak et al., in which an encapsulated compound in liposomes increased phototoxicity and decreased malignant cell motility following PDT in skin malignancies compared to healthy keratinocytes [83]. 

Among the metal-based nanoparticles used in psoriasis treatment, titanium dioxide, silver, gold, selenium, and platinum nanoparticles have garnered interest from the scientific community [84]. Although the knowledge about their use in PDT for psoriasis treatment is scarce, several studies have reported their beneficial application for topical administration in other epidermic cells. For instance, Feng et al. developed a folic acid-conjugated silica-coated titanium dioxide and assessed its biocompatibility in two cell lines: fibroblast cells (L929) and the human nasopharyngeal epidermoid cancer (KB) cells. After 24 h of incubation, the significantly increased uptake of folic acid-conjugated silica-coated TiO2 to L929 and KB cells was observed. After UV radiation, the system was non-toxic and led to the increased mortality of cells, confirming the photokilling ability of TiO2-based nanoparticles [85]. Regarding gold nanoparticles, Fereig et al. aimed to conjugate gold nanoparticles with chitosan nanoparticles and examined whether this hybridization would enhance the anti-psoriatic efficacy in vivo. As shown via a transmission electron microscope (TEM) and X-ray diffraction (XRD) analysis, the anti-inflammatory effect of the conjugate was evident from a lower spleen-to-bodyweight ratio and better histopathological skin condition compared to the other analyzed formulations [86]. Consistently, several other studies reported the potential option of gold-loaded nanoparticles for topical treatment [87,88,89]. Considering silver nanoparticles, David et al. aimed to evaluate the anti-inflammatory effects of these structures when conjugated with European black elderberry fruit extracts (*Sambucus nigra*—SN, in the Adoxaceae family). In vitro studies showed a decrease in cytokine production induced by UVB irradiation. In addition, in vivo studies revealed a reduction in edema and cytokine levels in paw tissues, suggesting their potential in the treatment of psoriasis lesions [90]. Taken together, the use of metal nanoparticles has the perspective to improve the efficacy of psoriasis treatment; however, more studies are required to draw further conclusions. 

## 5. Conclusions

After a thorough review of the literature, we suggest that PDT seems to have the potential to alleviate psoriasis. However, despite the ambiguous role of ALA-PDT and the fact that it has been extensively tested in psoriasis treatment, there is a gap in the research regarding other photosensitizers, the efficacy of which could have been promising. This highlights the need to evaluate more non-ALA photosensitizers on psoriatic in vitro and in vivo models, as well as in clinical trials. Another limiting factor observed in most of the reviewed studies was the side effects associated with PDT; however, we believe that this obstacle could be tackled by the appropriate utilization of nanotechnology.

## Figures and Tables

**Table 1:** A summary of in vivo studies and clinical trials evaluating the effect of PDT in psoriasis treatment.

Study	Photosensitizer	Way of Delivery	Wavelength	Treatment Parameters	Pre-Treatment with Drug	Number of Patients	Total Number of Treatment Sessions	Results	Side Effects	Reference
Treatment of psoriasis by topical photodynamic therapy with polychromatic light [24].	5-aminolevulinic acid (ALA, 10%)	Topical	600–700 nm	Light dose: 25 J/cm^2^ Dose rate: 70 mW/cm^2^	5 h	3	Max: - 3 times per week	Dithranol and topical PDT were comparable.	Burning sensations during irradiation	Boehncke et al. (1994)
The variable response of plaque psoriasis after a single treatment with topical 5-aminolaevulinic acid photodynamic therapy [25].	ALA (20%)	Topical	400–650 nm	Light dose: 2–16 J/cm^2^, Dose rate: 10–40 mW/cm^2^	4 h	22	Max: 12 Once a week	Thirty-five percent of patients’ psoriasis was cleared. 80 treatment sites: 14 cleared, 6 showed a 30–50% reduction in SEI score, 60 showed little or no improvement.	Stinging, tingling, burning sensations during and after illumination	Collins et al. (1997)
Improved response of plaque psoriasis after multiple treatments with topical 5-aminolaevulinic acid photodynamic therapy [44].	ALA (20%)	Topical	broad-band visible radiation	Light dose: 8 J/cm^2^ Dose rate: 15 mW/cm^2^	4 h	10	Max: 12 3 times per week	Eighty percent of patients responded to ALA-PDT. 19 treatment sites: 4 cleared, 10 responded but did not clear, 5 did not change.	Pain and discomfort (80% patients during treatment and 50% during and between treatments, respectively)	Robinson et al. (1999)
Systemic photodynamic therapy with aminolaevulinic acid induces apoptosis in lesional T lymphocytes of psoriatic plaques [45].	ALA (5,10 or 15 mg/kg)	Oral	Blue light, maximum at 417 nm	Light dose: for 5 or 10 mg/kg ALA: 1, 3, 6, 12, or 20 J/cm^2^ for 15 mg/kg ALA: 1, 2, 4, 8 or 10 J/cm^2^ Dose rate: 9–11 mW/cm^2^	1, 3 or 6 h	12	Max: 1 1 time per week	A 5 or 10 mg/kg ALA dose did not show improvement. 15 mg/kg ALA dose showed improvement.	Mild burning during light exposure	Bissonnette et al. (2002)
Lack of efficacy and tolerability of topical PDT for psoriasis in comparison with narrowband UVB phototherapy [46].	ALA (20%)	Topical	630 nm	Light dose: 10 J/cm^2^ Dose rate: 120 mW/cm^2^	4 h	4	Max: 12 1–3 times per week	SEI score reduced by 5% in 2 patients, 17% in 1 patient and was unchanged in one patient. These results were lower than the NB-UVB values.	Pain during treatment	Beattie et al. (2004)
Topical aminolaevulinic acid-based photodynamic therapy as a treatment option for psoriasis? Results of a randomized, observer-blinded study [47].	ALA (1%)	Topical	600–740 nm	Light dose: 5, 10, 20 J/cm^2^ Dose rate: 60 mW/cm^2^	4–6 h	29	Max: 12 2 times per week	Eight patients were excluded. Sixty-three treatment sites: 8 cleared, 53 from substantial to minimal improvement, 2 with no improvement.	Pain, stinging, burning during irradiation, lasting up to several hours	Radakovic-Fijan et al. (2005)
Clinical and immunohistochemical evaluation of psoriatic plaques treated with topical 5-aminolaevulinic acid photodynamic therapy [48].	Δ -ALA hydrochloride (20%)	Topical	630 nm	Light dose: 10–30 J/cm^2^ Dose rate: 20–315 mW/cm^2^	4–5 h	12	Max: 5 Once a week	Psoriatic plaques improved. The SEI score decreased.	Pain and discomfort during treatment	Fransson et al. (2005)
Disappointing results and low tolerability of photodynamic therapy with topical 5-aminolaevulinic acid in psoriasis. A randomized, double-blind phase I/II study [49].	ALA (0.1%, 1%, or 5%)	Topical	600–740 nm	Light dose: 20 J/cm^2^ Dose rate: 60 mW/cm^2^	4–6 h	12	Max: 12 2 times per week	Three patients were excluded. In the 0.1%, 1%, and 5% ALA-treated groups, the mean percentage improvement was 37.5%, 45.6%, and 51.2%, accordingly.	Pain and burning during and after irradiation	Schleyer et al. (2006)
A placebo-controlled randomized study on the clinical effectiveness, immunohistochemical changes and protoporphyrin IX accumulation in fractionated 5-aminolaevulinic acid-photodynamic therapy in patients with psoriasis [50].	ALA (10%)	Topical	600–750 nm	Light dose: 2 and 8 J/cm^2^ Dose rate: 40 mW/cm2	4 h + 2 h of dark interval	8	Max: 4 Once a week	Psoriatic lesions and plaques cleared, and plaque severity score decreased.	Burning and stinging during irradiation	Smits et al. (2006)
Topical 5-aminolaevulinic acid photodynamic therapy for intractable palmoplantar psoriasis [51].	ALA (20%)	Topical	630 nm	Light dose: 15 J/cm^2^ Dose rate: 30 mW/cm^2^	4 h	3	Max: 10 Once a week	The patients showed partial improvement in psoriatic lesions and plaques.	-	Kim et al. (2007)
Methylene blue mediated photodynamic therapy for resistant plaque psoriasis [52].	MB (0.1%)	Topical	670 nm	Light dose: 5 J/cm^2^ Dose rate: 565 mW/cm^2^	-	16	-	Sixteen patients showed improvement. Sixty-eight percent of the patients achieved a seventy-five percent reduction in severity score.	-	Salah et al. (2009)
Pulsed dye laser vs. photodynamic therapy in the treatment of refractory nail psoriasis: a comparative pilot study [52].	Methyl-aminolaevulinic acid (MAL)	Topical	595 nm	Light dose: 9 J/cm^2^ Dose rate: -	3 h	14	Max: 6 Monthly	Fourteen patients showed lower NAPSI scores. Both nail matrix and bed nail bed involvement cleared.	Slight pain during treatment	Fernandez-Guarino et al. (2009)
The effects of keratolytic pretreatment prior to fluorescence diagnosis and photodynamic therapy with aminolevulinic acid-induced porphyrins in psoriasis [53].	ALA (10%)	Topical	600–750 nm	Light dose: 10 J/cm^2^ Dose rate: 40 mW/cm^2^	6 h	10	Max: 6 Once daily	It was observed that psoriasis decreased, as well as clinical severity score.	Stinging, burning during irradiation	Kleinpenning et al. (2010)
A phase II placebo-controlled study of photodynamic therapy with topical hypericin and visible light irradiation in the treatment of cutaneous T-cell lymphoma and psoriasis [54].	Hypericin (0.05%, 0.1%, 0.25%)	Topical	590–650 nm	Light dose: 8 to 20 J/cm^2^ Dose rate: -	24 h	11	Max: 6 2 times per week	There was an improvement in skin lesions.	Mild burning and itching during treatment	Rook et al. (2010)
The Vitamin D Analog Calcipotriol Combined with Aminolevulinate-Mediated Photodynamic Therapy for Human Psoriasis: A Proof-of-Principle Study [33].	ALA (20%)	Topical	417 nm (blue light), 635 nm (red light)	Light dose: 10, 20 or 40 J/cm^2^ Dose rate: 100 mW/cm^2^	2 h	7	Max: 7 Twice daily	No clinical improvement in psoriasis was observed.	Stinging and pain during illumination	Maytin et al. (2012)
A retrospective analysis of real-life practice of off-label photodynamic therapy using methyl aminolevulinate (MAL-PDT) in 20 Italian dermatology departments. Part 1: Inflammatory and aesthetic indications [17].	MAL (160 mg/g)	Topical	635 nm	Light dose: 37 J/cm^2^ Dose rate: -	3–4 h	17	Max: 3.6 9.9 ± 5.6 days between treatments	Two patients experienced worsening psoriatic lesions. Three patients showed poor or no clinical improvement. Twelve patients showed a moderate or marked clinical response.	Pain or burning sensations during the treatment	Calzavara-Pinton et al. (2013)
Pyogenic granuloma in a patient with psoriasis successfully treated by 5-aminolevulinic acid photodynamic therapy: A case report [22]	ALA (20%)	Topical	633 ± 10 nm	Dose rate: 90 mW/cm^2^	3 h	1	1 session of ALA-PDT treatment followed-up weekly for 1 month	One week following the ALA-PDT treatment, the erosions had dried up, and the PG lesion was encrusted. No signs of recurrence were demonstrated 1 month after treatment	-	Liu et al., (2016)
Systemic ALA-PDT effectively blocks the development of psoriasis-like lesions and alleviates leucocyte infiltration in the K14-VEGF transgenic mouse [28].	ALA (65 mg/kg)	Injection	633 nm	Light dose: 108 J/cm^2^ Dose rate: 90 mW/cm^2^	2 h	6 (mice)	Max: 2 Weekly	ALA-PDT blocked the development of psoriasis-like lesions. The scores lowered.	-	Chen et al. (2017)
Anti-Psoriasis Effects and Mechanisms of A-(8-Quinolinoxy) Zinc Phthalocyanine-Mediated Photodynamic Therapy [41]. 1) ZnPc-F7-PDT effects on propranolol-induced psoriatic lesions in cavy 2) ZnPc-F7-PDT effects on IMQ-induced psoriatic lesions in Nu/Nu mice	1) ZnPc-F7 (1% or 5%)	Topical	630, 670 nm	Light dose: 14.15 J/cm^2^ Dose rate: 300–1500 mW/cm^2^	24 h	70 (cavies), n = 20	Max: 1	After 2 weeks of recovery, the ears exhibited no discernible abnormalities compared to normal animals. The histopathological traits remained, except for inflammatory cell infiltration	-	Liu et al. (2017)
	2) ZnPc-F7 (0.30, 0.60, 1.20 mg/kg)	Injection		Light dose: 19.10 J/cm^2^ Dose rate: -	6 h	70 (mice), n = 20	Max: 1	ZnPc-F7-PDT reduced the psoriatic symptoms caused by IMQ.		
ALA-PDT alleviates the psoriasis by inhibiting JAK signaling Pathway [27].	ALA (20%)	Topical	635 nm	Light dose: 12 J/cm^2^ Dose rate: 30 mW/cm^2^	4 h	Group (mice)	-	In IMQ-induced mice ALA-PDT reduced scaling, redness, erythema, scales, thickness and cumulative scores	-	Yi et al. (2019)
Photodynamic Therapy Combined with Dermabrasion in Cutaneous Squamous Cell Carcinoma Concomitant with Psoriasis [27]	Dermabrasion conjugated with ALA (20%)	Topical	635 nm He–Ne laser	Light dose: 100 J/cm^2^ Dose rate: 0.083 W/cm^2^ for 1200 s	5 h	1	four applications of PDT	The ulcer and plaque completely disappeared, and there were no obvious scars after treatment	-	Xu et al. (2019)
A Comparison of The Effects of Clobetasol 0.05% and Photodynamic Therapy Using Aminolevulinic Acid With Red Light in the Treatment of Severe Nail Psoriasis [36]	ALA (20%) and clobetasol propionate 0.05% ointment	Topical	630 nm (range 600–730 nm); red light-PDT	Light dose: 120 J/cm^2^ Dose rate: 200 mW/cm^2^	3 h	8	Every 3 weeks for 5 cycles	Six months after the last treatment session, the mean NAPSI scores in the nails treated with ALA-PDT were greater than those in the nails treated with clobetasol 0.05% ointment.	Slight pain during irradiation	Tehranchinia et al. (2020)
Effective topical treatments using innovative NNO-tridentate vanadium (IV) complexes-mediated photodynamic therapy in a psoriasis-like mouse model [43]	0.001% vanadium complex—PDT	Topical	Blue light 430 nm	Light dose: 0.1 J/cm^2^ Dose rate: -	4 h	imiquimod (IMQ)-induced psoriasis mouse model	30 min irradiation for 8 consecutive days	A higher phototoxicity index with non-toxicity under dark conditions, efficient skin morphological recovery according to the PASI score decrease in the percentage of IL-17A and IL-22 in the spleen.	-	Lin et al., 2022

## Data Availability

Not applicable.
